# Preoperative Risk Factors Associated with Increased Incidence of Postoperative Delirium: Systematic Review of Qualified Clinical Studies

**DOI:** 10.3390/geriatrics8010024

**Published:** 2023-02-07

**Authors:** Vlasios Karageorgos, Lior Mevorach, Melissa Silvetti, Federico Bilotta

**Affiliations:** 1 School of Medicine, University of Crete, 71003 Crete, Greece; 2 Department of Anesthesiology, Critical Care and Pain Medicine, Policlinico Umberto I Teaching Hospital, Sapienza University of Rome, 00185 Rome, Italy

**Keywords:** postoperative delirium, preoperative risk factors, diagnostic POD scales

## Abstract

Postoperative delirium (POD) is an acute alteration of mental state, characterized by reduced awareness and attention, occurring up to five postoperative days after recovery from anesthesia. Several original studies and reviews have identified possible perioperative POD risk factors; however, there is no comprehensive review of the preoperative risk factors in patients diagnosed with POD using only validated diagnostic scales. The aim of this systematic review was to report the preoperative risk factors associated with an increased incidence of POD in patients undergoing non-cardiac and non-brain surgery. The reviewed studies included original research papers that used at least one validated diagnostic scale to identify POD occurrence for more than 24 h. A total of 6475 references were retrieved from the database search, with only 260 of them being suitable for further review. Out of the 260 reviewed studies, only 165 that used a validated POD scale reported one or more preoperative risk factors. Forty-one risk factors were identified, with various levels of statistical significance. The extracted risk factors could serve as a preoperative POD risk assessment workup. Future studies dedicated to the further evaluation of the specific preoperative risk factors’ contributions to POD could help with the development of a weighted screening tool.

## 1. Introduction

Postoperative delirium (POD) is an acute alteration of mental state, characterized by reduced awareness and attention, occurring up to five postoperative days after recovery from anesthesia [1,2,3]. This neuropsychiatric disturbance seems to have an incidence ranging from 11% to 51% in older adults [4,5]. POD severely affects the postoperative recovery of patients, prolonging their hospital stay and increasing healthcare costs [6]. Furthermore, several studies suggest that POD is a ‘neurotoxic’ event possibly correlated with long-term effects such as postoperative cognitive dysfunction [4]. In 2017, the European Society of Anesthesiologists delivered dedicated guidelines that reported the need for routine monitoring using validated scales [7].

Multiple pathophysiological mechanisms underlying this phenomenon have been presented in the literature, with acetylcholine deficiency being one of the most established theories [8]. The central anticholinergic properties of anesthetic drugs, together with surgical stress, were considered to be the main contributors to POD’s pathogenesis [9]. However, several other possible risk factors have been suggested in the literature. These factors can be categorized as either modifiable or non-modifiable, and they can be detected before surgical procedures (pre-operative), during (intra-operative), or after them (postoperative) [10,11,12]. Several original studies and reviews have listed possible POD risk factors to be screened preoperatively. Some articles reported increased statistical association, while others calculated the odds ratio [13,14]. Interestingly, some articles claimed to report significant risk factors, although this significance was not verified in the statistical analyses. Such factors include sleep disruption at home, obstructive sleep apnea, total intravenous versus inhaled anesthesia, and general versus locoregional anesthesia [15,16,17,18,19,20]. The use of a risk stratification work-up that includes factors not clearly associated with POD can be substantially misleading and result in inappropriate patient selection or prescription of unnecessary drugs.

The available literature lacks a comprehensive report of the preoperative risk factors associated with an increased risk of POD based on a systematic review (SR) of clinical studies using only validated diagnostic scales. The aim of this SR is to report the preoperative risk factors associated with an increased incidence of POD in patients undergoing non-cardiac and non-brain surgery. The reviewed studies include original research studies that use at least one validated diagnostic scale to identify POD occurrence.

## 2. Methods

**Figure 1:**
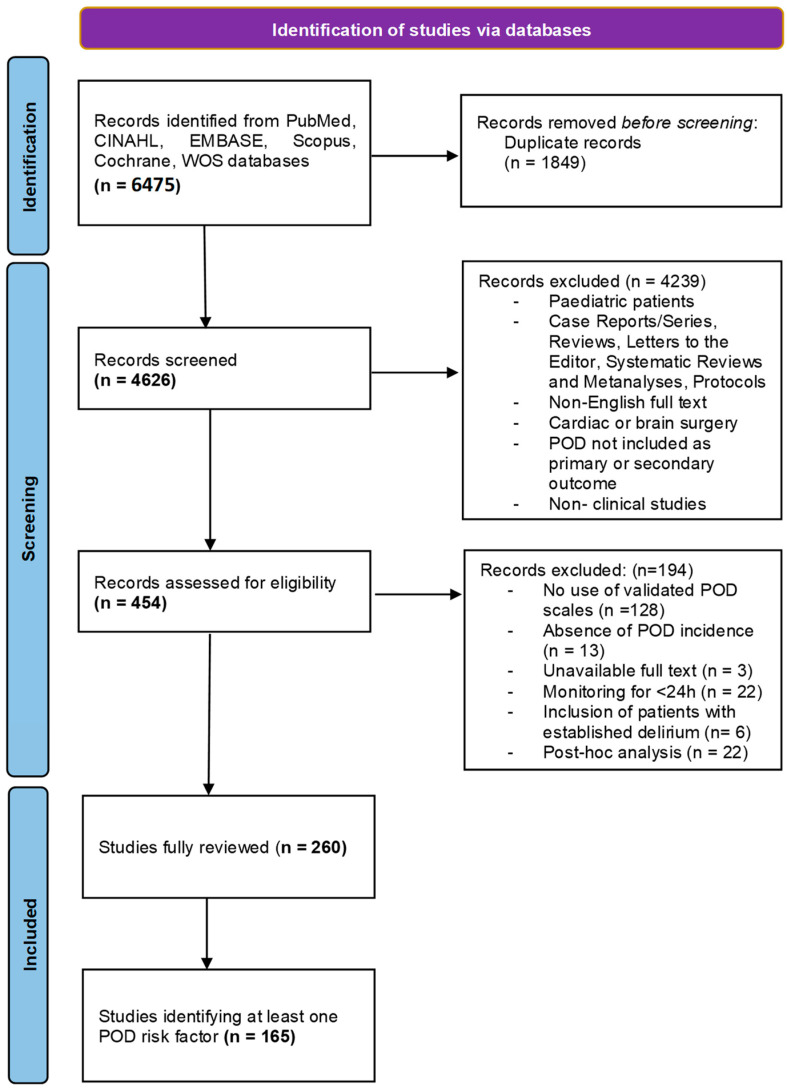
PRISMA flow diagram.

A systematic literature search was performed by assessing 6 databases in accordance with the PRISMA (Preferred Reporting Items for Systematic Reviews and Meta-Analyses) statement recommendations [21]. This SR was registered in the International Prospective Register of Systematic Reviews (PROSPERO registration number: CRD42021246906). It selectively included original studies that accomplished the postoperative monitoring of patients undergoing non-cardiac or non-brain procedures for longer than 24 h using validated scales [22]. In order to launch the related literature search, the following keywords were used: postoperative, delirium, confusion, post-anesthesia, and anesthesia recovery. Moreover, specific search strings were used in 6 scientific databases (Appendix A). A total of 6475 references were retrieved. The suitable studies included randomized clinical trials (RCTs), retrospective and prospective studies, case-control studies, and cohort studies. The other criteria included a population older than 18 years old, POD as the primary subject of the study, and English as the main manuscript language. Studies that did not include an original dataset (meta-analyses, reviews, commentaries, editorials, etc.), case-series presenting less than 5 cases, protocols of trials registered online, and reports published as abstracts were excluded. Of the reviewed studies, 260, which were published between 1987 and March 2021, were considered eligible (Figure 1).

The selected studies were thoroughly reviewed in order to identify those that reported preoperative risk factors associated with an increased incidence of POD. Apart from the preoperative risk factor extraction, the POD monitoring scale used was also recorded. As a secondary endpoint, the extracted POD risk factors are presented in a clinical record form (Table 1) that can serve as a literature-generated work-up for standardized preoperative risk stratification.

All the randomized studies that reported POD risk factors were evaluated using the Cochrane Collaboration’s risk of bias tool [23,24]. The risk of bias was assessed independently during the data extraction process.

## 3. Results

In 165 out of the 260 reviewed studies, one or more preoperative risk factors have been associated with an increased risk of POD. The types of POD monitoring scales used in the 165 studies are described. A total of 41 risk factors are identified and categorized into 7 groups: demographics, laboratory testing, comorbidities, cumulative scores, chronic therapies, habits, and type of surgery. Within each of these groups, individual risk factors are listed, reporting first those supported by the largest number of studies. For each variable, the lowest value associated with the statistical evidence, that is, with an increased incidence of POD, is reported as a threshold, and the odds ratio (OR) value or range indicated by all the relevant studies is mentioned.

### 3.1. Types of Scales Used

A single POD diagnostic scale is used in 110/165 (66.6%) studies, 48/165 (18.5%) used 2 diagnostic scales, and 3 or more scales are used in 7/165 (4.2%). The most frequently used diagnostic scale is the Confusion Assessment Method (CAM), which is included in 158/165 (95.7%); the Diagnostic and Statistical Manual of Mental Disorders (DSM) in 58/165 (35.1%); the CAM-Intensive Care Unit (ICU) in 38/165 (23.0%); the Delirium Rating Scale (DSR) in 20/165 (12.1%); the Delirium Observation Screening in 13/165 (7.8%); the Nursing Delirium Screening Scale (Nu-DESC) in 11/165 (6.6%); the Memorial Delirium Assessment Scale (MDAS) in 9/165 (5.4%); the International Classification of Disease (ICD) in 5/165 (3.0%); the Neelon and Champagne Confusion Scale (NEECHAM) in 5/165 (3.0%); the Delirium Symptom Interview (DSI) in 3/165 (1.8%); and the 4AT in 1/165 (0.6%).

### 3.2. Demographics

The included demographic risk factors are age, gender, and educational level. Advanced age is consistently and extensively reported as a POD risk factor in 88/165 (53.3%) of the studies selected as suitable for the present SR [25,26,27,28,29,30,31,32,33,34,35,36,37,38,39,40,41,42,43,44,45,46,47,48,49,50,51,52,53,54,55,56,57,58,59,60,61,62,63,64,65,66,67,68,69,70,71,72,73,74,75,76,77,78,79,80,81,82,83,84,85,86,87,88,89,90,91,92,93,94,95,96,97,98,99,100,101,102,103,104,105,106,107,108,109,110,111,112]. A higher incidence of POD is described in patients aged ≥60 years in 7/165 (4.24%) studies [44,45,108,109,110,111,112]. With increasing age, the POD risk becomes increasingly pronounced: patients aged 60–79 have an OR of 1.06–1.27 [54,55,56,57,58,59,60,61,62,63,64,65,66,67,68,73,101], while those aged ≥80 years old have an OR of 1.07–2.67 [27,103]. Gender, which is evaluated in 29/165 (17.5%) of the selected studies, is a possible predictive risk factor for POD [25,37,45,46,47,48,49,50,52,53,55,59,60,62,63,64,65,66,67,68,69,70,74,100,110,113,114,115,116]. In 24 studies [37,45,46,48,49,50,52,55,59,60,62,63,64,65,66,67,68,69,70,110,113,114,115,116], which recorded data from 7900 patients (5152 were male and 2748 female), reported no differences in the POD incidence between male and female patients. In 3 studies, female gender was associated with a higher risk of POD (up to an OR of 11.02) [25,47,53]. In 2 studies, male gender was associated with a higher risk of POD (up to an OR to 5.78) [74,100]. Educational level is considered a possible predictive risk factor for POD, as reported in 10/165 (6.0%) of the studies [26,50,54,55,61,62,65,70,100,117]. Patients with a lower educational level (<8 years) tend to have a high risk of POD [*p* = 0.001, OR = 3.27 (1.43–7.44)] [50,117].

### 3.3. Laboratory Testing

Among the laboratory testing risk factors are the plasma concentration of albumin, sodium, total bilirubin, blood glucose, calcium, hemoglobin, and pro-inflammatory markers (CRP, TNF-a, IL-6, C-reactive protein/albumin ratio). In total, 9/165 (5.45%) articles mention albumin as a possible POD risk factor [30,33,76,96,98,105,107,118,119]. Preoperative laboratory testing demonstrates that albumin is lower in patients with POD [33,118] and could possibly predict a higher risk when values are below 3.9 g/dL [119] (*p* < 0.002). The CRP levels are described as a possible preoperative risk factor in 5/165 (3.03%) studies [75,76,116,120,121]. Pre-operative C-reactive protein (CRP) is significantly higher (7.0 ± 15.2 mg/L, *p* < 0.017) in patients with delirium, and values above 7 mg/L are especially considered predictive of POD [116]. The hemoglobin levels are considered to be a possible risk factor for POD in 4/165 (2.42%) studies. In a total of 214 patients, a relevant hemoglobin level above 13.16 g/dL is associated with POD (*p* < 0.006) [76,98,107,122]. In 3/165 (1.81%) studies, blood glucose is demonstrated to be a possible risk factor for POD [76,99,107]. A blood glucose level >8.4 mmol/L is considered a predictive risk factor (*p* < 0.001, OR = 1.142) [99]. Another inflammatory marker, IL-6, is found in 3/165 (1.81%) studies to serve as a possible predictive marker for POD [98,121,123]. In one study, where 55/272 (20.2%) patients developed POD, higher levels of serum IL-6 are identified in the POD group preoperatively, further supporting that correlation (>17.9 pg/mL) (*p* < 0.0036, OR = 1.51). The sodium levels are found in 2/165 (1.21%) studies to be a possible high-risk factor of POD [74,107]. Values <135 mEq/L or >146 mEq/L are considered potential predictors of POD. In a study of 228 patients, 57 of whom (25%) developed POD, a sodium serum level between 136 and 145 mEq/L is revealed in 46/57 (80%) delirious patients. In 8/57 (14%) patients with POD, a serum sodium value over 145 mEq/L during the preoperative period is analyzed and found to be a sizeable risk factor in comparison to the non-POD group. In 3/57 (5.2%) patients with POD, a sodium serum level <136 mEq/L is identified to be highly associated with POD when compared to the non-POD group (*p* < 0.05) [107]. TNF-a is revealed in 2/165 (1.21%) studies to be a possible and considerable POD risk factor. A total of 79 patients who developed POD are demonstrated to have a higher TNF-a level (>8.2 nmol/L) than the non-POD patients (*p* < 0.018, OR = 1.43) [98,121]. Bilirubin is identified in one study as a potential risk factor, with 120/572 patients developing POD when the total bilirubin level is >18.00 µmol/L and the direct bilirubin is >5.1 µmol/L (*p* < 0.001, OR = 1.077) [76]. Serum calcium is found in one study to be a predictive factor for POD. Some 120/572 (20.9%) POD-positive patients have a serum calcium value <2.18 mmol/L, and hypocalcemia is revealed to be a predictive risk factor for POD (*p* < 0.004) [76]. The C-reactive protein/albumin ratio (CAR) is revealed to be a possible POD risk factor. In a study of 272 patients in which preoperative blood tests were conducted, the 55 patients who developed POD exhibit a higher CAR (>2.90), thus making it a relevant predictor (*p* < 0.001, OR = 3.04) [98].

### 3.4. Cumulative Indicators

Among the tested cumulative indicators found to be associated with an increased risk of POD are the MMSE score, ASA score, BMI, Charlson Comorbidity Index score, APACHE II, mini-cog score, Barthel Index, GDS, and CES-D fatigue status. The Mini Mental Scale Exam (MMSE), a diagnostic scale concerning cognitive function, is found to be a predictor of POD in 25/165 studies (15.1%) [20,35,41,42,48,50,51,54,60,62,63,65,66,70,71,74,86,97,98,105,109,114,116,124,125]. In particular, 145/450 (32.22%) patients with POD are demonstrated to have a lower level of MMSE (<17) compared to 47 patients without any POD event (*p* < 0.001) [35,86]. The American Society of Anesthesiology (ASA) physical status score is considered in 22/165 (13.33%) studies to be POD predictor [41,44,47,48,51,53,54,59,60,61,62,67,68,69,70,73,78,88,100,104,112,126]. In particular, 51/411 (12.4%) patients with POD are demonstrated to preoperatively have an ASA score below 2 and 32/411 (7.78%) patients to have an ASA score above 2 (OR = 2.21, *p* < 0.001) [88,112]. The Body Mass Index (BMI) is considered in 18/165 (10.9%) studies to be a predictor of POD [45,46,48,50,51,53,55,62,67,68,69,74,109,114,115,125,127,128]. In particular, 176/1061 (16.58%) patients with POD are demonstrated to have a BMI < 20 preoperatively (*p* < 0.05) [74,114]. The Charlson Comorbidity Index score (CCI) estimates the 10-year survival rates by examining several comorbidities, and it is considered in 8/165 (4.8%) studies to be a predictor of POD [30,51,72,74,83,85,86,109]. A higher CCI score (CCI ≥ 2) seems to be identified as a risk factor for POD development. The APACHE II score, a mortality index for ICU patients, is considered in 6/165 (3.6%) studies to be a predictor of POD [52,56,64,94,113,129], with 2 of the studies [94,129] identifying statistically significant correlation with POD development. The mini-cog score is a fast cognitive impairment screening test that can detect people with dementia. Scores ≤2 are considered to indicate impairment and to be a possible predictor of POD, as shown in 2/165 (1.2%) studies [30,130]. The Barthel Index for Activities of Daily Living is considered in 2/165 (1.2%) [30,74] studies to be a predictor of POD. Patients presenting with POD have lower Barthel Index values at a statistically significant level in both studies. The Geriatric Depression Scale (GDS) is considered in 3/165 (1.8%) studies to be a predictor of POD. An increased GDS score is associated with POD development in all three studies [46,74,114]. The CES-D fatigue status [78] is considered in 1/165 (0.6%) study to be a predictor of POD, with 30% of patients presenting with POD having a positive CES-D status preoperatively (*p* < 0.05).

### 3.5. Comorbidities

Various comorbidities are associated with an increased POD incidence. These include diabetes, cardiovascular conditions (coronary and vascular disease, arterial hypertension), cerebral conditions (depression, sensory impairment, history of stroke, cerebral vascular disease, sleep disorders), lung conditions (respiratory disease and pulmonary hypertension), and renal conditions.

Diabetes is considered in 12/165 (7.2%) [31,39,45,46,54,57,65,95,98,107,114,131] studies to be a potential predictor of POD. Only in 4/165 (2.4%) studies [31,39,95,131] is diabetes identified as a statistically significant risk factor (OR = 2.98). The remaining eight studies identified no or insignificant correlation between diabetes and POD.

Cardiovascular: Coronary and vascular disease is considered in 8/165 (4.8%) [26,31,45,57,95,98,114,132] studies to be a risk factor for POD development. Three of the studies identified statistically significant correlation between cardiovascular disease and POD development. Hypertension is considered in 8/165 (4.8%) [31,39,45,57,95,98,114,132] studies to be a potential risk factor for POD. Only in two studies (1.2%) [39,98] do researchers find statistically significant correlation between hypertension and POD development.

Cerebral: Depression is mentioned in one study 1/165 (0.6%) to be a risk factor for POD development at a statistically significant level (*p* < 0.001) [39]. Sensory impairment is considered in 2/165 (1.2%) [31,133] studies to be a potential risk factor for POD development. In one study [31], all types of sensory impairment are included, while the other focuses on hearing impairment [133]. Sensory deprivation is associated with an increased incidence of POD [134]. A history of stroke is identified as a potential risk factor for POD development in 5/165 (3%) studies [57,76,100,135,136]. Three of the studies suggest statistically significant correlation between prior strokes and POD (OR = 5.618) [76,100,135]. Cerebral vascular disease is highly correlated in two studies as a potential risk factor for POD development. A total of 99/462 (21.4%) patients with cerebrovascular disease developed delirium postoperatively [95,114]. Sleeping disorders are identified in 4/165 studies to be a potential risk factor for POD development [137,138,139,140]. The existence of obstructive sleep apnea syndrome is especially correlated with an increased incidence of POD (up to sixfold) in certain patient groups [137,138,139].

Renal failure is considered in two studies to be a potential risk factor for POD development. Only one study suggests that renal failure is associated at a statistically significant level with POD development (OR = 1.4) [39].

Lung conditions: Respiratory disease is considered in 3/165 (1.8%) studies to be a potential risk factor for POD [31,45,95]. However, only one study identifies statistically significant correlation [45]. Pulmonary hypertension is suggested in one study 1/165 to be a risk factor for POD (*p* < 0.001, OR = 1.8) [39].

### 3.6. Chronic Therapies

Among the chronic therapies, the following are included as risk factors: benzodiazepines [37,45,135,141], psychoactive drugs [135], B-blockers [98], and anticholinergics [104]. Benzodiazepine use is identified as a statistically significant risk factor in 4/165 (2.4%) studies (OR = 1.48–4.99) [37,45,135,141]. Psychoactive drugs are identified in one study to be a significant risk factor for POD development [135]. Treatment with beta-blockers is considered to be a risk factor for POD development in 1 study (*p* = 0.025) [98]. Anticholinergic drugs are considered to be a risk factor for POD development in 2/165 studies (1.2%) [104,142]. A special scale regarding the central anticholinergic effects of some drugs that was developed is known as the Anticholinergic Drug Scale (ADS). The ADS score is associated at a statistically significant level with POD development [104].

### 3.7. Habits

Among the habits considered to be possible risk factors are smoking [33,45,55,77,84,98,143], alcohol [25,31,33,39,45,55,57,77,84,132,133,144], and drug abuse [39]. Smoking is considered to be a potential risk factor for POD development in 7/165 studies [33,45,55,77,84,98,143]. Only one study identifies statistically significant correlation (*p* = 0.04) [55], with the rest of the studies finding no correlation [33,45,77,84,98,143]. Alcohol abuse is considered a predictor of POD development in 10/165 (6%) [25,31,33,39,45,55,77,84,132,133] studies. However, there is a big variance among the studies regarding the cut-off limit of the definition above. A total of 6 studies identify statistically significant correlation between alcohol abuse and POD development (OR = 2.3) [25,33,39,55,132,133], while 4 studies identify no correlation [31,45,77,84]. Drug abuse is considered a risk factor for POD development in a single study (*p* < 0.001) [39].

### 3.8. Type of Surgery

The type of surgery and indication criteria (elective or emergency) are strong predictors of POD. Intrathoracic and intra-abdominal [145,146] operations are associated with a higher incidence of POD compared to all the other surgical sites [107,144,145,146,147]. In the high-risk operations for POD, peripheral vascular procedures, urological procedures [OR = 4.03], spinal procedures [OR = 3.70], and orthopedic procedures [OR = 6.23] are included. [117,144]. Out of the orthopedic procedures, almost 1/3 patients undergoing total knee replacement and 1/4 patients undergoing hip arthroplasty develop POD [148]. Emergency surgeries are correlated with a high incidence of POD compared to elective ones, with up to 51% of patients admitted in an emergency setting developing POD [133]. As far as common surgical procedures are concerned, current clinical evidence regarding delirium is conflicting, with some observational studies reporting a lower risk of delirium with minimally invasive surgery, while others report no difference [53,149,150].

### 3.9. Risk of Bias Assessment

**Figure 2:**
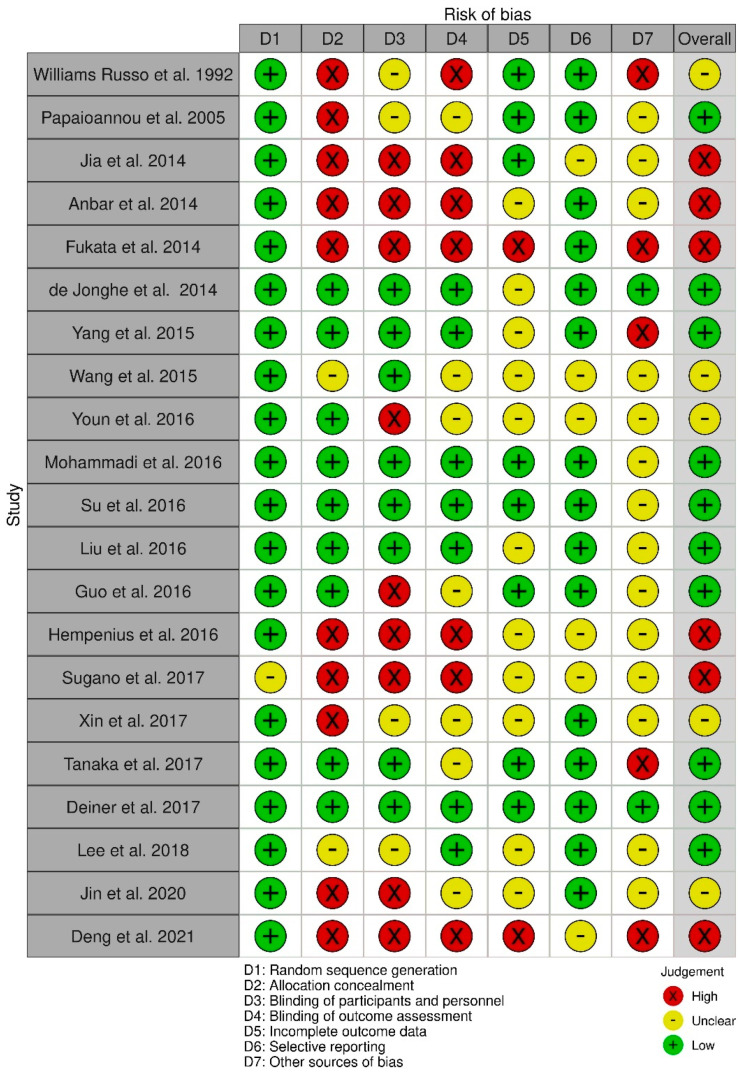

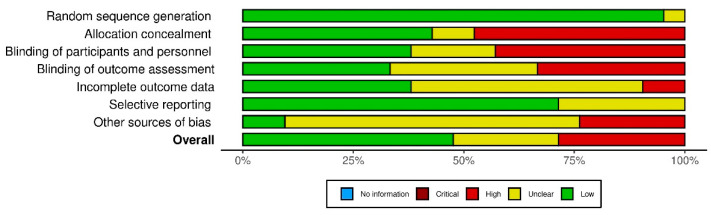
Risk of bias assessment of randomized studies [25,26,43,48,52,55,57,62,66,71,82,109,111,115,117,121,123,124,126,131].

All the randomized studies that report at least one risk factor included in the current study were evaluated for bias using the Cochrane Collaboration’s risk of bias tool. The overall assessment suggests an intermediate quality of data, attributed mainly to the limited reporting regarding the blinding of data (Figure 2).

## 4. Discussion

In this SR of clinical studies published online up to March 2021, only validated POD scales were used in adult patients undergoing non-cardiac or non-brain surgeries. Several preoperative risk factors associated with an increased incidence of postoperative delirium were identified. A large variety of diagnostic scales were used throughout the selected studies. The presented risk factors were categorized into seven groups: demographics (age, gender, educational level); laboratory testing (albumin, bilirubin, glucose, hemoglobin, CRP, TNF, IL-6, sodium); comorbidities (metabolic, cardiovascular, cerebral, lung, renal); cumulative indicators (MMSE score, ASA score, BMI, CCI score, APACHE II, mini-cog score, Barthel Index, GDS, CES-D fatigue status); chronic therapies (psychoactive drugs, beta-blockers, benzodiazepines); habits (smoking, alcohol abuse, drug abuse); and type of surgery and setting (intrathoracic, intraabdominal, peripheral vascular, orthopedic, urological, ambulatory, and emergency). Several studies in the field identified individual or lists of risk factors associated with POD development, although a specific preoperative risk assessment for POD is not sufficiently addressed yet, as a large number of them listed POD risk factors in the introduction or in the discussion part that were not supported by specific evidence [151,152,153,154,155,156,157,158,159,160,161,162,163,164,165,166,167,168,169,170,171,172,173,174,175,176,177,178,179,180,181,182,183,184].

This SR was conducted in accordance with the PRISMA guidelines [21], and it was based on using the literature search strategy proposed by the ESA task force on POD [7]. However, this approach may have restricted the studies we finally assessed due to the possible mismatch between the searched keywords and published data, as extracted by the algorithm used by the scientific library databases. It is of note that despite the large number of papers extracted, only 260 out of the 6475 (4%) fulfilled the criteria and used a validated diagnostic scale. The bias assessment suggested the intermediate level quality of the data overall. However, due to the high heterogeneity of study types included, it was not possible to perform a reliable quality assessment of all the studies using a single tool. This could be a limitation of our study. Finally, the selective inclusion of only original studies may have hindered us from identifying other potential POD contributors that have not been sufficiently studied yet or showed poor correlation due to the limited population size. These characteristics are, at the same time, possibly a limitation and quality criterion of the present SR.

In the present study, it is clear that among the variables associated with an increased incidence of POD, some have strong predictive value, as listed in Table 1. These variables should become part of every preoperative assessment and be reported with a specific focus on the evaluation of the POD risk. Interestingly, pain, which had been theorized to be a risk factor for POD and measured using the visual analogue pain scale (VAS), was tested in 3/165 (1.8%) studies but not found to be statistically significant in any of them [51,65,68]. The same applies for liver disease, where none of the studies identified statistically significant correlation with POD, with the exception of hepatic encephalopathy [45,185]. Dyslipidemia was evaluated in 4/165 (2.4%) studies to be a risk factor for POD [68,95,98,114], with no statistically significant correlation being identified, suggesting that other comorbidities that such patients have could be contributors to POD’s development. In a recent review by our study group, perioperative risk factors were identified [186]. This difference is attributed to the search criteria used during the literature screening, as well as to the fact that we only focused on preoperative risk factors but in a much wider timeline of published studies. We found some risk factors that are similar to those identified in the perioperative-focused research, as expected. However, there are some minor but substantial differences between the previous study and the current review. Despite the fact that the other research presents a similar subject, the evidence collected relies on independent processes (literature search, study selection, data extraction).

One of the study’s limitations is the great variance in the group characteristics in the reviewed studies, which did not permit us to perform a weighted analysis of the impacts of individual risk factors. Despite this, the use of a standardized data extraction form warrants the presentation of risk factors that are statistically associated with an increased incidence of POD, thereby mitigating the confounding impact of individual, procedural, or environmental-related characteristics.

## 5. Conclusions

The present SR provides a reliable and validated list of the preoperative risk factors associated with an increased risk of POD. Creating a screening tool using these risk factors should become a standard of care, and patients presenting an increased risk should be purposely treated throughout their hospitalization. Future studies should evaluate how to accurately identify high-risk patients and effectively minimize POD’s occurrence, as well as how to assess the influence of concurring risk factors on the perioperative clinical course.

## Figures and Tables

**Table 1:** Clinical record table including the risk factors correlated with POD development at a statistically significant level (cited in ≥2 studies).

Risk Factor		
Demographics		
	Age	≥60
	Gender *	Female
	Education	<8 years
Laboratory ^#^		
	Albumin	<3.9 g/dL
	CRP	>7 mg/L
	Hemoglobin	>13.16 g/dL
	Glucose	>8.4 mmol/L
	TNF-a	>8.2 nmol/L
	IL-6	>17.9 pg/mL
Cumulative scores		
	MMSE	<17
	CCI	≥2
	Mini–Cog	≤2
Comorbidities		
	Diabetes	Yes
	Stroke	Yes
Chronic therapies		
	Benzodiazepines	Yes
Habits		
	Alcohol abuse	Yes
Surgery		
	Emergency	Yes
	Intrathoracic, intrabdominal, Orthopedic, spinal, peripheral vascular	Yes
	Minimally invasive *	Yes

* Variables associated with controversial evidence ^#^ Variables associated with limited evidence. CRP = C-Reactive Protein, MMSE = Mini Mental State Examination, CCI = Charlson Comorbidity Index.

## Data Availability

Data is contained within the article.

## Appendix A. Full List of Literature Search Strings

1. **PubMed:** (delirium OR delirious OR confusion OR disorientation OR bewilderment) AND (postoperative OR “postoperative” OR post intervention OR “post intervention” OR “post-surgical” OR postsurgical OR “post-surgery” OR post surgery OR “anesthesia recovery” OR “anesthesia recovery” OR “anesthesia recovery period”[- Mesh] OR post anesthesia OR “post anesthesia” OR “post anesthesia”)
2. **EMBASE:** (((‘delirium’/exp OR delirium OR delirious OR ‘confusion’/exp OR confusion OR ‘disorientation’/exp OR disorientation OR bewilderment) AND (‘postoperative complication’/exp OR postoperative OR ‘post-operative’ OR ‘postoperative period’/exp OR post-intervention OR ‘post intervention’ OR ‘post-surgical’ OR postsurgical OR ‘post-surgery’ OR post-surgery OR ‘anesthesia recovery’/exp OR ‘anesthesia recovery’ OR ‘anesthesia recovery’/exp OR ‘anesthesia recovery’ OR post anesthesia OR ‘post anesthesia’ OR ‘post anesthesia’)) OR ‘postoperative delirium’/exp) AND ([cochrane review]/lim OR [systematic review]/lim OR [meta-analysis]/lim OR [randomized controlled trial]/lim OR ‘observational study’ OR ‘case study’)
3. **CINAHL:** (delirium OR delirious OR confusion OR disorientation OR bewilderment) AND (postoperative OR “postoperative” OR post intervention OR “post intervention” OR “post-surgical” OR postsurgical OR “post-surgery” OR post-surgery OR “anesthesia recovery” OR “anesthesia recovery” OR post anesthesia OR “post anesthesia” OR “post anesthesia”) Limiters—Publication Type: Case Study, MetaAnalysis, Randomized Controlled Trial, Systematic Review OR ((delirium OR delirious OR confusion OR disorientation OR bewilderment) AND (postoperative OR “postoperative” OR post intervention OR “post intervention” OR “post-surgical” OR postsurgical OR “post-surgery” OR post-surgery OR “anesthesia recovery” OR “anesthesia recovery” OR post anesthesia OR “post anesthesia” OR “post anesthesia”)) AND (observational study OR observational research) OR (MH “delirium” OR MH “confusion+”) AND ((MH “postoperative complications”) OR (MH “postoperative period”)) Limiters—Publication Type: Case Study, MetaAnalysis, Randomized Controlled Trial, Systematic Review OR (MH “delirium” OR MH “confusion+”) AND ((MH “postoperative complications”) OR (MH “postoperative period”)) AND ((observational study OR observational research))
4. **COCHRANE:** #1 ((delirium OR delirious OR confusion OR disorientation OR bewilderment) AND (postoperative OR “postoperative” OR post intervention OR “post intervention” OR “post-surgical” OR postsurgical OR “post-surgery” OR post-surgery OR “anesthesia recovery” OR “anesthesia recovery” OR post anesthesia OR “post anesthesia” OR “post anesthesia”)):ti,ab,kw 1829 #2 MeSH descriptor: [Delirium] explode all trees 765 #3 MeSH descriptor: [Confusion] explode all trees 899 #4 #2 OR #3 899 #5 MeSH descriptor: [Postoperative Period] explode all trees 5872 #6 MeSH descriptor: [Postoperative Complications] explode all trees 39635 #7 MeSH descriptor: [Anesthesia Recovery Period] explode all trees 2031 #8 #5 OR #6 OR #7 43861 #9 #4 AND #8 326 #10 #1 OR #9 1853
5. **SCOPUS:** TITLE-ABS (delirium OR delirious OR confusion OR disorientation OR bewilderment) AND TITLE-ABS (postoperative OR {post-operative} OR post intervention OR {post intervention} OR {post-surgical} OR postsurgical OR {post-surgery} OR post-surgery OR {anesthesia recovery} OR {anesthesia recovery} OR post anesthesia OR {post anesthesia} OR {post anesthesia}) AND TITLE-ABS ({systematic review} OR {case series} OR {randomized controlled trial} OR rct OR {meta-analysis} OR metanalysis OR {observational study})
6. **WEB OF SCIENCE:** TITLE-ABS (delirium OR delirious OR confusion OR disorientation OR bewilderment) AND TITLE-ABS (postoperative OR {post-operative} OR post intervention OR {post intervention} OR {post-surgical} OR postsurgical OR {post-surgery} OR post-surgery OR {anesthesia recovery} OR {anesthesia recovery} OR post anesthesia OR {post anesthesia} OR {post anesthesia}) AND TITLE-ABS ({systematic review} OR {case series} OR {randomized controlled trial} OR rct OR {meta-analysis} OR metanalysis OR {observational study})
